# “It’s given me confidence”: a pragmatic qualitative evaluation exploring the perceived benefits of online end‐of‐life education on clinical care

**DOI:** 10.1186/s12904-021-00753-y

**Published:** 2021-04-13

**Authors:** Deidre D Morgan, Caroline Litster, Megan Winsall, Kim Devery, Deb Rawlings

**Affiliations:** 1 Palliative and Supportive Services, College Nursing and Health Sciences, Flinders University, South Australia Adelaide, Australia; 2 Research Centre for Palliative Care, Death and Dying, Flinders University, South Australia Adelaide, Australia

**Keywords:** Online education, End‐of‐life care, Terminal care, Allied health professional, Nurses, Physicians, Self confidence

## Abstract

**Background:**

Hospital admissions for end-of-life care are increasing exponentially across the world. Significant numbers of health professionals are now required to provide end-of-life care with minimal training. Many health professionals report they lack confidence to provide this care, particularly those in acute hospital settings. This study explored the perceived benefits of online education on health professionals’ capacity to provide end-of-life care.

**Methods:**

This qualitative study adopted a pragmatic approach. Thirty semi-structured interviews were conducted with allied health professionals, nurses and doctors who had completed a minimum of three End-of-Life Essentials online education modules. Interviews were held on line and face-to-face, audio-recorded and transcribed verbatim. Demographic data were also collected. Three major themes and one minor theme were constructed from the data using inductive thematic analysis.

**Results:**

Themes were (1). Perceptions of preparedness to provide end-of-life care, (2). Shifts in approaching end-of-life discussions and (3). Motivation for engagement with online modules. Participants reported validation of knowledge and improved confidence to have end-of-life discussions with patients, carers and team members. They also noted improved ability to recognise the dying process and improved conversations with team members about patient and carer needs. Videos portraying a novice and then more able end-of-life discussions were particularly valued by participants. Modules provided practical guidance on how to engage in discussions about the end of life and care needs. Participants were self-motivated to improve their knowledge and skills to enhance end-of-life care provision. Continuing professional development requirements were also a motivator for module completion.

**Conclusions:**

This study explored health professionals’ perspectives about the perceived benefits of online education modules on their clinical practice. Module completion enhanced participant confidence and self-reported improved competence in end-of-life care provision. Findings build on existing research that supports the valuable role online education plays in supporting confidence and ability to actively engage with patients, carers and colleagues about provision of end-of-life care; however, self-report cannot be used as a proxy for improved clinical competence.

## Background

End-of-life care provision for those with incurable and chronic diseases is applicable throughout the illness trajectory in all care settings. Hospital admissions for end-of-life care are increasing exponentially worldwide [1–3]. People with advanced chronic conditions like Chronic Obstructive Pulmonary Disease (COPD) or dementia often experience acute health changes that trigger repeat hospitalisations, ultimately requiring quality end-of-life care [4, 5]. Subsequently, many health professionals, without specialist end-of-life care training, are being called upon to make rapid end-of-life care decisions across the illness trajectory. Health professionals of all disciplines report feeling isolated and ill equipped to provide end-of-life care, both specialist and generalist. They describe limited confidence and preparedness to engage in this important area of clinical practice [6–8]. Care in acute hospitals also has a curative approach, focusing on effecting timely and safe discharges. Adapting clinical care for people with illnesses that cannot be cured requires health professionals to adopt a different mindset and approach to clinical practice [9].

The World Health Organization (WHO), recognizing the growing worldwide demand for, yet limited access to end-of-life care, urges all member States to strive to incorporate training about end-of-life as an integral part of clinical education [10]. The WHO identifies three levels of training - basic training (e.g. in-services, continuing professional education across all care settings), intermediate training (e.g. for those routinely working with people at the end of life) and specialist training which focuses on advanced symptom management and care needs. Multidisciplinary end-of-life health professional education has been incorporated into formal fee paying undergraduate and postgraduate courses in a number of countries such as Australia, United States of America, Norway and the United Kingdom [11–14]. In Australia, discipline specific end-of-life professional development training (medical, nursing and allied health) is also promulgated via discipline peak bodies, government and non-government organisations [15–17]. Undergraduate and postgraduate education while important, is unlikely to keep up with the need to build end-of-life care capacity in the hospital workforce overall. Access of quality evidence-based resources about end-of-life care is one form of education that can inform multidisciplinary end-of-life clinical practice at the basic WHO level [8, 10, 18].

Online or e-learning continues to grow in popularity as an accessible mode of health professional education [19]. It offers a cost-effective mode of continuing professional development [20–22] that mitigates the need to travel long distances [23]. It is likely the demand for online-mediated education will increase in light of the COVID-19 pandemic [24]. This study sought to explore health professionals’ perspectives about the perceived benefits of End-of-Life Essentials (EOLE) online education modules on their clinical practice as it related to end-of-life care provision [25]. End-of-Life Essentials is an Australian government funded website that was established for health professionals who work in an acute setting. It provides free peer reviewed information and resources for multidisciplinary health professionals. The EOLE website is comprised of ten modules which can be accessed freely online (https://www.endoflifeessentials.com.au/). End-of-Life Essentials is not a formal education program with assessable study requirements. Participants can create a toolkit for their own reference that includes videos, podcasts, action checklists related to professional development goals an individual may set for themselves sourced from modules on completion. Modules cover a range of topics for health professionals in hospital settings about working with people at the end of life. They do not provide specific clinical advice, rather they provide general education about issues around dying and focus, for example, on enabling conversations about end of life.

## Methods

### Study design

Study design was informed by a clinical pragmatist philosophy and worldview [26]. Pragmatism does not commit itself to a specific epistemology or reality, rather, methods are selected on their relevance to the issue under investigation. A pragmatist approach views thoughts and actions as interlinked and that consequences of actions influence future choices [27]. Adopting a pragmatic worldview [28] enabled a focus on what works or doesn’t work in clinical practice around end-of-life care provision and perceived benefits of EOLE online education modules on health professionals’ behaviour and choices around that care. A qualitative evaluation was conducted with multidisciplinary health professionals who had enrolled in EOLE and completed a minimum of three online education modules. A minimum of three modules ensured that participants would be able to speak to module content. For the purposes of this study, end-of-life care is defined as “the services provided around the immediate time of death, or broadly as the approach adopted once it is clear that a health condition is likely to lead to death in the relatively near future” [28].

### Sampling and recruitment

Potential participants were eligible for this study if they were health professionals who had registered to access EOLE modules and had self-reported completion of a minimum of three modules. Purposive sampling by profession was employed to ensure a diversity of views. An email to introduce the study and invite registered EOLE users to participate was sent to a subset of doctors, nurses and allied health clinicians. The majority of registered EOLE users work in acute hospitals where end-of-life care is not routinely provided in the same way it is in specialist palliative care settings. All emails were targeted by discipline and tailored to that specific group i.e., separate invitations were sent to doctors, nurses and allied health professionals. The study was also promoted via newsletters, social media and professional groups and at a conference booth. Those who responded to the invitation and met the inclusion criteria were sent an email with a link to a digital consent form and a short online survey collecting background demographics. Verbal consent was also verified at time of interview. Sixty-eight health professionals responded but five later declined an interview due to time constraints. Twenty-seven did not respond to a second interview invitation. Four were ineligible because they were not working in a clinical role, or had no clinical experience while two had completed less than three modules. Participant identity was known only by the interviewer and not the other researchers.

### Data collection

Qualtrics online survey software, held on a secure university platform, was used to collect and store demographic data. Thirty semi-structured, in-depth interviews were conducted face-to-face, both in their place of employment [2], by telephone [27] or online via Zoom [1] between May 2019 and January 2020 with consented participants. Participants who took part via phone were not asked to disclose their location. Interviews took between 19.49 and 68 min. A $25 gift card was offered in recognition of time given for interview participation. Development of a semi-structured interview guide was informed by a pragmatic worldview [28] and clinical experience. It was used to elicit information regarding impact of the EOLE modules on health professionals’ end-of-life care practice (Table 1). Consistent with an inductive approach, interview questions were refined early in the data collection process to incorporate emerging issues [29].

**Table 1:** Semi-structured interview guide

**Introductory questions**
Can you tell me a bit about your work setting and your role? How long have you been working in your current position?
**Transition questions**
Could you tell me how often you care for a person who has end-of-life care needs?
How well prepared have you felt to care for a person with end-of-life care needs?
What has helped you develop the clinical skills to provide care for people with end-of-life needs? *(Probes – undergraduate, postgraduate, on the job training? Organisational support?)*
So given your access to those resources/training what made you choose to do the End-of-Life Essentials education modules/Tool Kit? *(Probes – personal professional development, workplace requirement?)*
**Key questions**
Do you recall any of the themes or content of the modules/toolkit? Is there one module or modules/toolkit in particular that has been the most helpful for you in your everyday clinical practice? *(Probes – Why is that? Which aspects were most helpful/least helpful?)*
What is the most challenging thing when working with a person who has end-of-life needs? *(Probe – In what ways have the modules/toolkit influenced your clinical care of those with end-of-life needs?)*
How have the EOLE modules /toolkit affected your confidence in identifying patient needs? *(Probe – In what ways has this influenced your ability to identify patient needs?)*
In what ways have the EOLE modules /toolkit influenced your ability to raise end-of-life discussions with other team members/patients and carers?
Could you describe the most significant change to your clinical practice since completing the EOLE education modules/toolkit?
Can you talk with me about any barriers/challenges you have faced when using the knowledge and skills you have learned from the EOLE modules/toolkit?
Conversely what supports do you have in your workplace to implement what you have learnt from the EOLE modules/toolkit?
Can you talk about how your organisation approaches patient end-of-life care needs?
Would you recommend the EOLE education modules to colleagues? *(Probes - Can you tell me why you answered in this way?)*
If you could change anything about the EOLE eLearning modules/toolkit, what would it be?
**Ending questions**
Of all the things we discussed today, which one is the most important to you?
Is there anything else we should talk about but didn’t?

 Audio recordings were transcribed verbatim and participants were given the opportunity to verify transcripts as a true and accurate record of the interview. One participant made minor amendments to their transcript. Ethical approval was obtained (Flinders University Human Research Ethics Committee ref 7012).

### Data analysis

An inductive thematic analysis approach was employed to construct themes iteratively from the data [30]. NVivo V.12 (QSR International Pty Ltd) was used to organise, code and explore the data. Initial codes were constructed by two researchers (CL, MW) in vivo using participant phrasing and language. The two coding sets were then transferred to excel sheets to enable refining of coding, led by an experienced qualitative researcher (DM). Inductive data saturation informed the themes’ development from refined codes via an iterative process [31]. Consensus agreement was reached on themes and subthemes (DM, CL, MW) and final themes were verified by the research team (DM, CL, MW, KD, DR). This study complied with COREQ guidelines where possible [32].

## Results

### Participant Characteristics

A total of 30 interviews were conducted with seven doctors, 16 nurses, one occupational therapist, three social workers, one physiotherapist, one paramedic, and one radiographer. All participants came from Australia, except one from New Zealand. Participant ages were recorded in ten-year blocks and ranged from 20 s through to 60 + years. Years of work experience ranged from one to 37 years. Just under two thirds of participants worked in acute hospital settings and other settings where people with end-of-life care needs are seen infrequently. Table 2 describes participant characteristics while Table 3 reports the numbers of people with end-of-life care needs routinely cared for by participants.

**Table 2:** Participant Characteristics

	Doctors	Nurses	Allied Health	TOTALS	
**Gender**					
Male	2	1	1	4	13.3 %
Female	5	15	6	26	86.7 %
**Age (years)**					
20–29	1	0	1	2	6.65 %
30–39	2	3	0	5	16.65 %
40–49	2	2	2	6	20 %
50–59	2	7	2	11	36.7
60+	0	4	2	6	20 %
**Years’ Experience**					
5 years or less	1	4	2	7	23.35 %
6 to 10 years	3	5	0	8	26.65 %
11 to 15 years	1	4	0	5	16.65 %
16 to 20 years	1	2	3	6	20 %
21 years or more	1	1	2	4	13.35 %
**Primary work area**					
Acute	4	9	2	15	50 %
Sub-acute, including ambulatory care	1	4	3	8	26.65 %
Other	1	3	0	7	23.35 %
**Region**					
Metropolitan	6	11	6	24	80 %
Rural	1	4	1	6	20 %

**Table 3:** Frequency of people cared for with end-of-life care needs

	Doctors	Nurses	Allied Health	Totals	
Daily	2	6	4	12	40 %
Weekly	3	4	2	9	30 %
Fortnightly - monthly	0	3	0	3	10 %
Less often/infrequently/unclear	2	3	1	6	20 %

Three overarching themes and one minor theme constructed from the data analysis are summarised in Table 4. Detailed descriptions of themes, subthemes and supporting quotes highlighting participant perspectives are provided.

**Table 4:** Perceived benefits of online end-of-life learning on clinical care – Themes and subthemes

Theme	Subtheme
1. Perceptions of preparedness to provide end-of-life care	1.1 Affirmation of knowledge improves confidence 1.2 Recognising when end of life is approaching 1.3 Strengthening interprofessional teamwork 1.4 Currency of real-life scenarios in video format
2. Shifts in approaching end-of-life discussions	2.1 Proactively initiating end-of-life discussions 2.2 Being present and available 2.3 Listening and using silence 2.4 Enhancing discussion about end-of-life care needs
3. Motivation for engagement with online modules	3.1 Self-motivated learning to improve my skills 3.2 Flexibility of engagement for learning 3.3 Meeting professional requirements and recommendations
4. Perceived educational needs that remained unmet	4.1 What didn’t work and what could be improved

Participants described perceived benefits of the EOLE online modules on their clinical practice with people with end-of-life care needs. In line with a real-world pragmatic approach, themes articulate the practical perceived benefits of online learning on clinical care.

### Perceptions of preparedness to provide end‐of‐life care

#### Affirmation of knowledge improves confidence

Participants reported that evidence-informed module content validated and reinforced their existing clinical knowledge, improving confidence in their clinical skills. Clinical applicability of module content was noted to fit into existing ward practices and encouraged individual and team reflection about ways to improve end-of-life care. Reduced avoidance and increased confidence to engage in difficult conversations with patients and family was reported to be one of the most significant skills participants developed post module completion. Knowledge gleaned from modules enhanced participant ability to actively engage with and respond to patient and family emotions around pending death. While some participants reported greater confidence, others noted this was a work in progress.

*“Just until I started doing the modules I tended to just go, “Oh just a minute I’ll come back with someone else who’s a bit more equipped than me, has more experience.” And I probably would still do that until I gained more experience, but I don’t fob the question off so quickly anymore”* (1, Nurse).

*“…a lot of that sort of confidence and knowledge that we are on the right path, and that’s just there to keep going you know, with that especially I think the modules said it is all right to talk about dying and also not to avoid the difficult conversations and another thing*… *I think in doing modules myself, it gives me an idea that they can fit in with the work that we are trying to do on the ward… But what I’m finding with doing the modules is that it fits very much with our own resources that we use at work.”* (11, Nurse).

#### Recognising when end of life is approaching

Participants reported module content was readily translatable into everyday clinical practice which prompted participants to reflect on patterns of readmission that indicated patients were approaching the end of life. These patterns related primarily to people with chronic diseases (e.g. COPD, dementia). Participants also reported enhanced recognition of the terminal phase of care and associated bodily changes.

*“…because it had videos to go with it had people talking, real life basically scenarios that I could relate to because I saw that every day in my practice where you know someone would come in and they’ve got pneumonia but then they’ll go home but then they’ll come back two months later. Now I recognise that patients coming back and forth back and forth is not – what’s the word I’m looking for- it’s not normal you know what I mean? So, whether it’s two years, five years, six months who knows, I now recognise that that’s why they’re coming back and forth, because we’re so complacent because we’re so used to them. That was a big learning curve for me was knowing that they are already in the end-of-life stage.”* (3, Nurse).

#### Strengthening interprofessional teamwork

 Participants noted an increased openness with colleagues regarding their own need for support to initiate end-of-life conversations. They also reported heightened confidence to raise and contribute to team discussion about end-of-life issues following module completion, supporting a more consistent team approach to determining patient end-of-life goals of care. The use of medical, nursing and allied health video case scenarios expanded participant perspectives and understanding about the scope and role of other health professionals in end-of-life care. It also extended understanding of patient care needs and options for referral to help meet those needs.

*“I guess about what was going on and so the multidisciplinary team were a bit disjointed and you know lacking in you know in communication so I did have a bit of a chat with the doctors and the rest of the team, and we actually have you know a family meeting with the patient present there so that everybody was on the same page, and I did actually, I facilitated that meeting and sort of used all those communication skills that we were just speaking about before… Yes, I think I feel more confident that I have you know knowledge that I can contribute regarding this particularly this tuition or topic and so I think I feel a lot more comfortable having discussions…”* (22, Social Worker).

#### Currency of real‐life scenarios in video format

Videos had a lasting impact on participants, demonstrating how to have honest, albeit difficult conversations with patients about the end of life. Participants highlighted the value of a range of clinical scenarios which provided valuable context and non-verbal nuances not found in written texts. A recurrent theme related to the value of the ‘Novice-Able’ format of the teaching videos which presented an inexperienced clinician interaction that did not engage with patient needs and then re-presented in a more constructive way. The absence of judgement around a Novice conversation was highly regarded, and an Able response noted as something to work towards. Critique of videos by participants noted that actors were often young and videos were somewhat artificial at times.

*Well, I remember one of the things I really liked, was that they would do the same scenario from the view, with a novice, person doing it so not done that way and then the same scenario with an expert, person doing it. and I thought that that was a really good way to teach it without making people whose skills might not be very advanced feel bad about that…”* (17, Doctor).

### Shifts in approaching end‐of‐life discussions

#### Proactively initiating end‐of‐life discussions

 Initiating end-of-life conversations was viewed with new importance by participants. They described how the online training enabled them to initiate these discussions more readily in a respectful and kind way. Learning to recognise and raise end-of-life concerns with patients, carers and colleagues was considered a courageous process which had been difficult prior to module completion. Participants highlighted increased ability to respond to uncertainty associated with patient responses. One participant noted that module content gave her permission to sit with and explore patient concerns without crossing her professional boundaries.

*“I found that online training particularly useful just for initiating conversations and starting that dialogue and also talking with my peers because that’s a big thing a lot of people don’t talk about palliative care and end-of-life care so much until that’s actually happening so I been able to make myself a bit more open to my peers and get that support where I need it when it comes to the situation.”* (4, Nurse).

*“…one patient in particular who I saw over a period of time … was really struggling to come to terms with expecting that she was end of life, she was quite young… and I just had the opportunity one day to say you know “what do you think is happening in your body? You know what’s your stance of what’s going on?” and that just kind of opened a big door if that makes sense? And I do think … maybe I would have done that anyway, without the modules, but I do think they just helped me to know that that’s okay.” (23, Physiotherapist)*.

#### Being present and available

Online education eased participant anxiety about having insufficient skills to answer patient questions about end-of-life care. Participants described modifying their interactions to be more present and available to patient and family vulnerabilities. Module content challenged provision of care in a mechanical way and improved participant comfort with conducting difficult discussions about poor prognosis and dying. Participants described increased ability to accommodate differences required in communication styles with different age groups and cultures facing the end of life following module completion.

*“… you know we all feel the same, and we all don’t know how to say things or use the right word sometimes and you’re going to come across a lot of different emotions and a lot of culturally different emotions too and just knowing, I guess you’ve just got to just- and be there and present- something I did learn: be there and present for each person…”* (8, Nurse).

*“Yeah, I do remember actually, not long after doing one of the modules… someone was crying and it had said in the training about, you know, allowing them- of course they are going to process their grief and, you know, perhaps you know- I might have said, “It’s okay,“ but I think in the training it said, of course it’s not okay, they’re dying, but to comfort them or support them in the knowledge that they know that they’re dying… it was very soon after I had done the [modules], I had supported the family and the person dying when they were grieving about their prognosis.*”(10, Nurse).

#### Listening and using silence

When talking with people facing the end of life, participants often felt pressured to come up with the ‘right answer’ to concerns. Module content guided participants to validate how patients and families were feeling about their situation instead. A recurrent theme was that of learning to listen and not be afraid to leave silences in conversations. Time constraints of busy clinical wards made it difficult to spend time actively listening to patients, despite its acknowledged importance. However, participants noted that module content reinforced that listening to what patients really need may only take a few extra minutes, so not something to avoid.

*“… it was like black and white questions without a black and white answer… the things that I found really good about the modules have been that we don’t know and it’s okay but just to validate and to have that conversation. And people just need to – you need to be – I need to listen and respond. And not handle it but to validate yes to validate the person and do the best that I can.”* (1, Nurse).

*“Yes, but certainly that’s one of those case scenarios they use silence to listen to the patients and let her talk. I’ve used that – I really take that note and use that quite a lot. That’s probably one of the ones I took away, [in] my own practice.”* (5, Nurse).

#### Enhancing discussion about end‐of‐life care needs

Participant engagement in discussion about care needs was enhanced by module completion. Of particular benefit were the suite of key phrases to use when discussing end of life. Phrases were easily understood by patients and helped participants identify end-of-life needs and priorities and perceived to put patients at ease. Participants also noted module videos encouraged them to be more explicit about dying and death in a tactful but forthright way, and to facilitate a space where people could ‘lean into’ distressing emotions.

*“It did make me think about making me a bit more explicit with people and so I’ve tried to do that. Bearing in mind that you have to kind of judge the person. So some people don’t like to hear death and dying and terminal and so sometimes you have to kind of judge whether they want to hear those words or whether you need to be a bit vaguer at times but I have tried to be a bit more explicit about it. Because I know that the evidence would suggest that most people would rather a degree of not bluntness, but forthrightness I guess, tactful forthrightness.” (17, Doctor)*.

*“I think as my role as a Med Reg now, compared to being a resident, you have to do it a lot more. And it’s just…how would I put this, because you do it enough, it doesn’t take as long, because you’re not fumbling for your words…although it’s still very personal for patients and for family, … you’re more direct about what you’re saying. I think that’s what you sometimes need in end of life… Like, sometimes you just actually say, look, it’s not going to go well. We don’t expect a great recovery. That’s us being pragmatic. Obviously, things can change, but we- in this case, we don’t think it’s going to change and that’s why we need to have this conversation now. And I think sometimes when you say, I know it’s very early to be discussing something like this, but we want to make sure that if something does happen, especially like with some of the patients that most probably have very good goals of care, and have full resuscitation, we always just say- we just want to make sure we’re taking your interests and your thoughts into this process early on.” (27, Doctor)*.

### Motivation for engagement with online modules

#### Self‐motivated learning to improve my skills

Participants valued lifelong learning. Online learning enabled them to update and refresh their clinical knowledge and enhanced confidence. Motivation to be better prepared to provide end-of-life care led to module completion for some participants as this could be daunting. While some reported seeing people at the at the end of life infrequently in their normal clinical caseload, others noted increasing numbers in their care. Those working in non-specialist palliative care settings sought additional knowledge to enhance care provision across the dying trajectory. A focus on improving communication for team members with patients and families, irrespective of discipline and/or clinical experience was a recurrent theme for module engagement.

*“… I always feel as though there’s more to learn, and I think when you’ve been in a position for a lengthy period of time you have to be aware of the fact that new knowledge comes along, new ways of looking at same old knowledge. You know it’s important, not only for the job but important for your own mental stimulation to keep up with it.”* (21, Occupational therapist).

*“It just kind of piqued my interest and it seems to have provided solutions for some difficult circumstances I’ve found myself in. So I just used this as a self-help tool … I found it quite useful, to the point nearly where I kind of – well I did look into maybe retraining in palliative care but I haven’t quite made that commitment yet.”* (2, Doctor).

#### Flexibility of engagement for learning

Free, evidence-based information that that was engaging and accessible at a time and place that suited was highly valued. While professional development was encouraged by most workplaces, participants noted opportunity to do so was limited in work time. Some completed modules completion at home, even when education was part of a paid award. Self-directed learning could be completed within a short time frame and participants noted they could return to modules as needed. One rural participant highlighted the benefits and value of e-learning.

*“Look I think for us particularly rurally, e-learning is about the best way that we have to access learning, particularly self-directed learning where we’re trying to improve our knowledge in areas that we’ve traditionally not been skilled for.”* (26, Paramedic).

*“I did it all in my own time. I find it very difficult – if I’m in theatre I can’t concentrate on patient care and doing courses but it was easy to do. Each module may have been an hour or so. This time of year, cold and dark evenings – you can sit down and do one every night or so.”* (2, Doctor).

#### Meeting professional requirements and recommendations

Participants described a range of external factors that affected module completion including supervisor or colleague recommendation or a performance appraisal requirement. Clinical educators reported using module content for lectures and other workplace professional development forums. No participant reported compulsory EOLE module completion, although professional development was compulsory. Professional bodies’ recognition of Continuing Professional Development points for EOLE modules provided an external motivator for participation.

*“I’ve used them in all three sections of my job… I’ve used them for Acute Care Hospitals, I’ve used them at my, you know, in education as a Clinical Nurse Educator and I’ve used them in my university lectures but I’ve been using it to educate staff, you know and nurses, undergraduate nurses…”* (5, Nurse).

### Perceived educational needs that remained unmet

#### What didn’t work and what could be improved

Participants offered suggestions on how to improve the online learning experience. Some noted the volume of educational information could be challenging to negotiate.

*“…as I got into it and went bit down a bit of a rabbit hole to tell the truth. I think some of the links were broken. […] I kept on going to multiple links and almost getting lost.” (10, Nurse)*.

The introduction of discipline specific topics and modules pertaining to bereavement, grief and loss, clinician self-care and resilience were also suggested.

*“I know that a lot of the modules will have … doctors’ in a role play… the video, they’ll have doctors or they’ll have allied health… I think because social work is so important in the end-of life-conversations and end of life processes and things that it would be nice to have… some roles played in there around social workers facilitating some of those conversations or facilitating … communicating with the person and the family. I think it would be helpful specifically from my practice” (22, Social worker)*.

The potential for an interactive capacity on modules for questions and answers along with virtual face-to-face forums was also raised.

*“I would love to do a face like- you know like we have a study day, we did one on delirium and dementia which was so informative […] I just cannot find where you can go and have like this study day and you all go and you talk and you share your experiences, because that is so vital, sharing experiences you’ve been in. […] I couldn’t care if you paid for it and you could go to it. That would be the ultimate.” (8, Nurse)*.

## Discussion

End-of-Life Essentials online educational resources are designed to inform health professionals who work in acute hospitals about general principles of care of people at the end of life, the basic educational level as defined by the WHO [10]. This qualitative evaluation explored perceived benefits about EOLE online resources. Study findings identified three major themes and one minor theme regarding the perceived benefits and challenges of online education modules.

A key finding in this study was the participants’ self-reported enhanced confidence to initiate or engage in discussions about end-of-life care with patients, carers and colleagues following module completion [33]. Improving confidence to engage in end-of-life care discussions is important as patient satisfaction with care has been found to improve when health professionals engage in end-of-life discussions in a timely manner [34, 35]. Enhancing health professionals’ ability to initiate these conversations is vital as patients don’t always speak up about approaching end of life, assuming the health professional will initiate this [35, 36] Engaging in end-of-life care with patients has been identified as one of the most challenging but most important considerations for health professionals when caring for people at the end of life. [37–39]. Although communication skills training is included in most health professional education, minimal time is spent on how to broach end-of-life conversations [7, 21, 40]. The modules provided a framework by which health professionals could proactively respond to patient and carer needs. Participant responses in our study are consistent with other research that identifies a need for communication skills training, how to best care for family carers and improvement in multidisciplinary team communication about patient and carer needs [33, 37]. Since its launch, more than 20,000 health professionals have registered with EOLE with over 36,000 module completions, indicating an interest in this general level of education about end-of-life care. Self-directed learning is considered an adaptive response, empowering people to work in complex changeable workplaces [41, 42]. However, research also that suggests informal self-directed learning competence requires active cultivation [43].

Participants in this study repeatedly talked about the important but challenging scenario of engaging carers in discussions about a loved one’s end-of-life care needs. A carer’s intimate knowledge of a loved one can provide staff with valuable knowledge about a person’s deterioration that can inform patient care. Yet carers note that they are not always given opportunity to raise this with health professionals [44]. It is critical therefore, that health professionals develop a baseline competency and confidence to initiate end-of-life discussions when appropriate with patients and carers. Participants in this study self-reported an enhanced ability to draw on a suite of responses gleaned from the modules that enabled them to better manage challenging questions or emotional responses from patients and families. Some indicated it gave them the courage to initiate end-of-life discussions. Health professionals also recognise this but report needing additional training [14, 21, 33]. Importantly, our findings build on existing studies that report additional training, including use of suites of responses such as question prompt lists, improves end-of-life discussions for health professionals, patients and families [45, 46].

A recurrent theme in our study was the value of the novice-able video suite of medical, nursing and allied health communication exemplars that show an inexperienced clinician-patient exchange followed by a more skilful exchange. Mentoring and observing experienced professionals communicate effectively about end-of-life issues supports growth of health professionals end-of-life communication skills [47–49]. Videos provide a flexible accessible opportunity for modelling of bedside interactions when face-to-face mentoring may not be readily available in an acute setting where provision of end-of-life care is not routine everyday practice [50]. As noted earlier, many health professionals are ill-prepared to provide end-of-life care; however, consistent with our findings, targeted simulation-based education has been found to improve clinician preparedness for end-of-life care provision [15]. While the novice-able videos are not simulation-based training, our participants reported changes in clinical practice similar to those who had completed simulation-based training [51]. These included reflecting on personal challenges with end-of-life communication, then practising new ways of having end-of-life discussions informed by the video modelling. However, further investigation is required to evaluate how reflexivity informed by online training truly impacts clinical care.

### Implications for clinical practice

The need for health professionals to develop end-of-life care skills in all health settings may be greater now than in any other time in history. A burgeoning ageing and multimorbid population is now joined by mushrooming numbers of people with COVID-19 who are extremely unwell and may die, or are fearful of contracting what may be a life-limiting illness. Growing evidence supports the value of mentoring, modelling and simulation-based learning for health professionals in end-of-life communication [35, 52]. Online asynchronous education offers a pragmatic flexible adjunct for face-to-face learning that may improve health professional confidence in communicating with patients, carers and team members about end-of-life care needs and provision.

### Limitations & future areas for research

Interviews were conducted at single point in time so findings do not record long-term change in clinical practice. Self-report cannot be used as a proxy for improved clinical competence [53] and there is insufficient research to establish effectiveness of EOLE and other e-learning programs on patient outcomes [22]. However, findings support emerging research reporting improved confidence following education [47, 48, 54, 55]. Participants were a motivated self-selecting group with an interest in end-of-life care; therefore, results do not capture responses of health professionals who do not consider providing end-of-life care within their remit. This study identified organisational issues but only captured individual self-reported responses. Societal and organisational factors have been identified as crucial in supporting self-directed learning competency [41]. Future research could evaluate systems factors that influence the individual’s delivery of patient care and forms the foundation for further evaluation of end-of-life care competency pre and post module completion [56].

## Conclusions

This study explored health professionals’ perspectives about the perceived benefits of online education modules (EOLE) on their clinical practice. While efforts have been made to integrate end-of-life education into curricula internationally, experienced health professionals still express a need for clinical professional development to support end-of-life care delivery. While findings build on existing research supporting the valuable role online education plays in supporting confidence to actively engage with patients, carers and colleagues about end-of-life care provision, it is not possible to determine the effect on patient outcomes nor learner competence.

## Data Availability

Data can be made available to bona fide researchers upon request as long as it complies with Flinders University Human Research Ethics Committee requirements.

## Abbreviations

EOLE: End-of-Life Essentials
COPD: Chronic Obstructive Pulmonary Disease

## Declarations

### Author qualifications

Deidre Morgan: BAppSc(OT), PGCert (Palliative Care), Grad Dip(pastoral counselling), MClSc(OT), PhD

Caroline Litster: BSocSc(Hons)

Megan Winsall: BSc, Grad Dip Public Health

Kim Devery: BSocSc(Hons)

Deb Rawlings: BSc(Hons), MPH
